# Whole genome sequencing of *Clarireedia* aff. *paspali* reveals potential pathogenesis factors in *Clarireedia* species, causal agents of dollar spot in turfgrass

**DOI:** 10.3389/fgene.2022.1033437

**Published:** 2023-01-05

**Authors:** Bochra Amina Bahri, Rajiv Krishna Parvathaneni, Willis Turner Spratling, Harshita Saxena, Suraj Sapkota, Paul L. Raymer, Alfredo D. Martinez-Espinoza

**Affiliations:** ^1^ Department of Plant Pathology, University of Georgia, Griffin, GA, United States; ^2^ Institute of Plant Breeding, Genetics and Genomics, University of Georgia, Griffin, GA, United States; ^3^ Department of Crop and Soil Sciences, University of Georgia, Griffin, GA, United States

**Keywords:** *Clarireedia paspali*, PacBio and Illumina technologies, gene prediction, protein structure prediction, genome annotation, phylogeny, pathogenicity, effectors

## Abstract

Dollar spot is one of the most damaging diseases in turfgrass, reducing its quality and playability. Two species, *Clarireedia monteithiana* and *C. jacksonii* (formerly *Sclerotinia homoeocarpa*) have been reported so far in the United States To study the *Clarireedia* genome, two isolates H2 and H3, sampled from seashore paspalum in Hawaii in 2019 were sequenced *via* Illumina paired-end sequencing by synthesis technology and PacBio SMRT sequencing. Both isolates were identified as *C*. aff. *paspali*, a novel species in the United States Using short and long reads, *C*. aff. *paspali* H3 contained 193 contigs with 48.6 Mbp and presented the most completed assembly and annotation among *Clarireedia* species. Out of the 13,428 protein models from AUGUSTUS, 349 cytoplasmic effectors and 13 apoplastic effectors were identified by EffectorP. To further decipher *Clarireedia* pathogenicity, *C*. aff. *paspali* genomes (H2 and H3), as well as available *C. jacksonii* (LWC-10 and HRI11), *C. monteithiana* (DRR09 and RB-19) genomes were screened for fifty-four pathogenesis determinants, previously identified in *S. sclerotiorum*. Seventeen orthologs of pathogenicity genes have been identified in *Clarireedia* species involved in oxalic acid production (*pac1*, *nox1*), mitogen-activated protein kinase cascade (*pka1*, *smk3*, *ste12*), appressorium formation (*caf1*, *pks13*, *ams2*, *rgb1*, *rhs1*) and glycolytic pathway (*gpd*). Within these genes, 366 species-specific SNPs were recorded between *Clarireedia* species; twenty-eight were non-synonymous and non-conservative. The predicted protein structure of six of these genes showed superimposition of the models among *Clarireedia* spp. The genomic variations revealed here could potentially lead to differences in pathogenesis and other physiological functions among *Clarireedia* species.

## Introduction

Turfgrass is a valuable commodity worldwide and in the United States It is used in home lawns, golf courses, sport fields and recreational lands and shown to improve groundwater recharge, soil erosion and soil carbon sequestration (Held and Potter 2012). The turfgrass industry contributes more than 822,848 jobs and has a total economic impact of $57.94 billion annually in the United States (Haydu et al., 2006). However, dollar spot, the most common and damaging disease in turfgrass, represents a significant risk for the expansion and the economic sustainability of this commodity. It has a worldwide distribution and can infect most cultivated turfgrass species. The disease is typically controlled by repeated fungicide applications. Because of high demand in turfgrass aesthetic quality and playability, mitigating dollar spot disease represents a major expense for the turfgrass industry (Smiley et al., 2005) and fungicide applications, resulting in environmental safety issues including the emergence of fungicide-resistant dollar spot strains (Putman et al., 2015).

The disease was at first attributed to the ascomycete *Sclerotinia homoeocarpa* F.T. Bennett (Bennett, 1937). However, taxonomic classification of the causal agent remained unresolved until 2018. Based on three DNA markers (rDNA internal transcribed spaces ITS region, calmodulin CaM and DNA replication licensing factor Mcm7), Salgado-Salazar and colleagues (Salgado-Salazar et al., 2018) placed the causal agent in a new genus, *Clarireedia*, as a member of the family Rutstroemiaceae rather than the Sclerotiniaceae family. Their study described four species for the genus *Clarireedia*, *C. homoeocarpa*, *C. bennettii*, *C. jacksonii*, and *C. monteithiana*. *C. jacksonii* and *C. monteithiana* were the two species reported in the United States and the most prevalent worldwide species, infecting cool- and warm-season turfgrasses, respectively. *C. homoeocarpa* and *C. bennettii* were isolated on warm-season turfgrasses and were restricted to the United Kingdom (Salgado-Salazar et al., 2018). Recently, a new species, *C. paspali*, was reported on seashore paspalum (*Paspalum vaginatum*) in different provinces of China (Hu et al., 2019). This species is characterized by the presence of an intron at the 3′-end of the small subunit ribosomal ribonucleic acid region, which genetically separated *C. paspali* from *C. jacksonii*, and *C. monteithiana*. However, within *C. paspali*, a group named *C*. aff. *paspali*, which did not present this intronic region, was genetically differentiated (Hu et al., 2019). Typically, the molecular identification of *Clarireedia* pathogens and their differentiation at the species level requires conventional Sanger sequencing using universal primers. In their study, Salgado-Salazar et al. (2018) identified twenty-eight, twenty, and eight species-specific SNPs differentiating *C. monteithiana*, *C. jacksonii*, *C. homoeocarpa*, and *C. bennettii* in the ITS, CaM and Mcm7 sequences, respectively (Salgado-Salazar et al., 2018). However, recently Groben et al. (2020) were able to quickly diagnose *Clarireedia* from field samples using a quantitative real-time PCR assay. In addition, a co-dominant cleaved amplified polymorphic sequence assay differentiating *C. monteithiana* from *C. jacksonii* was recently developed (Stackhouse et al., 2021). Beside variations at the molecular level (Powell and Vargas 2001; DeVries et al., 2008), diversity in vegetative compatibility groups (VCGs) and mating-type locus were also reported in *Clarireedia* spp. (Powell and Vargas 2001; Viji et al., 2004). Overall, the clonal reproduction of the pathogens supported the low level of genetic diversity observed (Powell and Vargas 2001; Viji et al., 2004; DeVries et al., 2008). However, some *Clarireedia* studies provided evidence that heterokaryosis and random mating increased genetic variability in nature (Hsiang and Mahuku 1999; Kessler et al., 2018; Salgado-Salazar et al., 2018) and stimulated research interest in exploring the genomic diversity, population dynamics, and structure.

So far, few research studies have investigated the pathogenicity in *Clarireedia* and knowledge in turfgrass-*Clarireedia* interactions are currently lacking. Although, *C. jacksonii* and *C. monteithiana* have been predominantly isolated in United States fields from cool-and warm-season turfgrasses, respectively (Salgado-Salazar et al., 2018), artificial cross inoculations showed no host specificity for dollar spot infection (Aynardi et al., 2019; Sapkota et al., 2020). In fact, under artificial inoculations, no variability in host range and only differences in virulence level were observed between *Clarireedia* isolates (Chakraborty et al., 2006; Steketee et al., 2017). Several virulence factors in plant pathogenic fungi were shown to contribute to a successful infection. Oxalic acid was detected in pure cultures of *Clarireedia* spp. (Venu et al., 2009) as well as in infected turfgrass tissues (Orshinsky et al., 2012a; Rioux et al., 2020) and was suggested to be an important pathogenicity factor as it is in *S. sclerotiorum*, the closest well-characterized fungal pathogens (Bolton et al., 2006). This compound was identified to play a role in symptom development and host colonization (Rioux et al., 2020). Additionally, glycosyl hydrolase enzymes and serine proteases, previously reported as pathogenicity factors in several fungal pathogens with a wide host range (Rowe and Kliebenstein 2007; Li et al., 2010), were identified in *Clarireedia* spp. transcripts and up-regulated in dollar spot infected creeping bentgrass (Orshinsky et al., 2012a). Further studies are still needed to decipher the full pathogenicity pathways of *Clarireedia* species at the molecular and genomic levels.

Over the past two decades, the development of next-generation sequencing (NGS) technologies and the contemporary advances in computational biology and bioinformatics, have boosted species diagnosis and scientific discoveries. Several studies in plant pathogens have highlighted the use of NGS technologies in providing high throughput species/strain level characterization and investigating the changes in population dynamics (Hubbard et al., 2015; Malapi-Wight et al., 2016; Chalupowicz et al., 2019). By coupling Illumina with PacBio approaches, combining short-read and long-read sequencing datasets, scientists were able to reach a higher level of whole genome sequencing and genome assembly for several plant pathogen species (De Miccolis Angelini et al., 2019; Porto et al., 2019). Typically, a well-curated and assembled reference genome for mapping NGS reads is crucially needed to facilitate pathogen identification, resolve taxonomy of dollar spot species, and perform phylogenetic and comparative genomic analysis across strains and pathogen species. Whole genome assembly is also important to understand genomic structure of pathogen species and to infer important phenotypic characteristics (such as virulence and fungicide resistance) and predict pathogen dynamics over time. To date, draft genomes for ten *Clarireedia* isolates have been generated: HRI11, HRS10, LWC-10, MB-01, SH44, SE16F4, CPB17, and LT30 were classified as *C. jacksonii*; while DRR09 and RB-19 were identified as *C. monteithiana* (Green et al., 2016; Salgado-Salazar et al., 2018; Crouch et al., 2021; Sapkota et al., 2021). Eight out of the ten *Clarireedia* genomes assembled were only sequenced using Illumina technology. The maximum genome sizes for *C. jacksonii* and *C. monteithiana* were reported for HRI11 with 43.35 Mbp sequenced with Illumina and PacBio (Green et al., 2016) and DRR09 with 48.70 Mb sequenced with Illumina (Crouch et al., 2021), respectively. Overall, the genomes currently available for *Clarireedia* spp. are fragmented (with >231 scaffolds), and most genomes lack information on genome features, and are not annotated. In addition, there are no comprehensive studies on the genomes and the genomic diversity in *Clarireedia* spp. Annotation of the genomes and identification of the genetic variation between the different *Clarireedia* species responsible for dollar spot are essential for understanding the epidemiology, pathogenicity, evolution, and host specialization of the pathogens and for improving turfgrass disease management strategies.

The overall goal of this study is to genetically and molecularly characterize *C*. aff. *paspali*, a new species of the *Clarireedia* genus causing dollar spot in turfgrass. This study aims to gain insight into shared genomic features among *Clarireedia* species and between the *Clarireedia* species and the closely related fungal species of the Sclerotiniaceae and Rutstroemiaceae families. The objectives were to 1) present the first draft genome of *C*. aff. *paspali*, 2) compare the genome features of *C*. aff. *paspali* with other *Clarireedia* species, 3) perform a phylogenetic analysis to understand the relationship among *Clarireedia* species and between *Clarireedia* and its close fungal relatives, and 4) compare at the genomic and protein levels the genome of *C*. aff. *paspali* with other *Clarireedia* species for a range of possible determinants involved in pathogen morphogenesis and pathogenesis. In this study, *S. sclerotiorum* and *R. sydowiana* were used as representatives of the Sclerotiniaceae family and the Rutstroemiaceae family, respectively.

## Materials and methods

### Sample collection, DNA extraction and pathogen identification

Plugs of seashore paspalum displaying dollar spot symptoms on leaves were sampled by a sod producer in Hawaii in September of 2019 (lat. 21.42827; long. -158.02432). The symptoms were characterized by white to straw-colored lesions, tip dieback, and irregular sunken patches in the turf stand (Sapkota et al., 2020). Two isolates, H2 and H3, were isolated and purified from the cores following the protocol described by Sapkota et al. (2020). Briefly, seashore paspalum leaves with dollar spot symptoms were surface sterilized with 10% bleach and 80% ethanol for 2 min each, rinsed with sterile autoclaved water three times, cut into 1-to-2 cm pieces, plated on potato dextrose agar (PDA) plates, and incubated at room temperature. White fluffy mycelium growth was visible 24-hr after incubation. The actively growing mycelium was transferred to another PDA plate and this step was repeated three times to obtain pure cultures. For each isolate, a seven-day old mycelial culture was scrubbed from the PDA medium and DNA extraction was performed using cetyl trimethylammonium bromide (CTAB) method (Doyle and Doyle 1987). The internal transcribed spacer (ITS) region of ribosomal DNA, calmodulin (CaM) gene, elongation factor 1 (EF) gene, and b-tubulin (TUB) gene of the two isolates were amplified using ITS5-ITS4 (White et al., 1990), CMD5-CMD6 (Hong et al., 2005), EF1F-EF1R (Carbone and Kohn 1999), and Bt2a- Bt2b (Glass and Donaldson 1995) primer sets, respectively. Two isolates, *C. jacksonii* (DS-CB, also known as DS3) and *C. monteithiana* (DS-SP, also known as DS8) sampled in Griffin, GA in 2019 from bentgrass and seashore paspalum, respectively, were also added as references (Sapkota et al., 2020). Amplicons were analyzed by 2% agarose gel electrophoresis, Sanger sequenced, and blasted against the NCBI database. Sequence alignment with ClustalW and comparison of ITS and EF regions from H2 and H3 isolates with *Clarireedia* reference isolates sampled from seashore paspalum from Hu et al. (2019) (reference isolates BH3, BH1, HK1, and SZ1 of *C. jacksonii*, *C. monteithiana*, *C*. aff. *paspali*, and *C. paspali*, respectively), was also performed under Mega X (Kumar et al., 2018). Species-specific single nucleotide polymorphisms (SNPs), identified by Salgado-Salazar et al. (2018), were also investigated in the aligned ITS sequences.

### Pathogenicity test

Creeping bentgrass (*Agrostis stolonifera*) cv. A-1/A-4, zoysiagrass (*Zoysia matrella*) cv. Zorro, bermudagrass (*Cynodon dactylon*) cv. Princess, and seashore paspalum (*Paspalum vaginatum*) cv. SeaStar turfgrasses were used for the pathogenicity test of H2 and H3 isolates. Plants were grown in Kord nursery pots (3” × 3”) filled with Metro-MIX 852 RSI professional growing mix (Sun Gro Horticulture). Treatments consisted of H2-inoculated pots, H3-inoculated pots, and uninoculated control pots for each turfgrass species. Each treatment consisted of three replications for a total of thirty-six pots. H2 and H3 inoculum was prepared using sterile, water-soaked mixtures of wheat, barley, and oat seeds. One-week old H2- or H3-colonized Potato Dextrose Agar (Difco) plugs were independently added to grain mixes in 500 ml Erlenmeyer flasks. Mycelia of H2 and H3 were allowed to infect and proliferate through grain mixes for 2 weeks. Five infected grain seeds were then added to all corresponding treatment pots, and five uninfected grain seeds were added to each control pot. After inoculation, pots were arranged randomly in nursery trays and trays were covered with Mondi propagation domes that were sprayed with sterile water to increase and maintain humidity. Trays were then placed in a growth chamber (Conviron, Pembina, ND, United States) and covered with black plastic bags that had been sprayed with sterile water. The bags were removed after 2 days in the growth chamber, and disease symptoms were evaluated in the following days. The growth chamber used for the pathogenicity test was programmed to operate at 23°C and 90% relative humidity with a 12h/12 h photoperiod (day/night). Disease was visually scored using a severity scale (% turf area blighted) with an index of 0–10 (0 = asymptomatic; 1 = 1%–10% symptomatic tissue; 5 = 41%–50% symptomatic tissue; 10 = 91%–100% symptomatic tissue) (Viji et al., 2004; Aynardi et al., 2019). DS-SP *C. monteithiana* isolate sampled in 2019 in seashore paspalum in Georgia United States (Sapkota et al., 2020) was also added as reference in the pathogenicity test.

### Library construction and DNA sequencing

Seven-day old mycelial cultures of H2 and H3 isolates were scrapped from the PDA medium and sent on dry ice to BGI Americas Corporation (https://www.bgi.com/us/) for DNA extraction, library construction and DNA sequencing with NGS technologies. High-quality genomic DNA (gDNA) was extracted from both isolates using CTAB protocol (Doyle and Doyle 1987). DNA quantification and purity were evaluated using Qubit™ Fluorometric quantitation (Thermo Fisher Scientific, Inc., Waltham, MA, United States) and NanoDrop reading, and 0.6% TAE agarose gel was run to check the DNA quality in order to meet the required standards for sequencing (sample concentration≥50 ng/ul and sample purity A260/280 at 1.8–2.0).

For one of the two isolates (H3), a 20 K library was constructed for PacBio SMRT sequencing. A large amount of high-quality DNA (20 µg) was used for the long-read library preparation, per the manufacturer protocols (PacBio, Menlo Park, CA, United States). SMRTbell Express Template Prep kit (PacBio, Menlo Park, CA, United States) was used for library template preparation. Briefly, g-TUBE (Covaris, Inc., United States) and AMPure PB beads (Beckman Coulter Inc. United States) were used to shear the gDNA into ∼20-kb fragments and for fragment size selection, respectively. To remove the single-stranded ends and repair DNA damage, the sheared gDNA was treated with ExoVII enzyme. Then, single-molecule real time (SMRT) hairpin adapters were ligated to the ends of the double-strand fragments polished with T4 DNA Polymerase. Subsequently, the adapter-ligated SMRTbell template was digested with ExoIII and VII enzymes for the removal of failed ligation products. The library was again purified with AMPure PB beads, quantified using Qubit™ Fluorometric quantitation and quality checked using a 2100 Bioanalyzer (Agilent Technologies, Inc., Santa Clara, CA, United States). Sequencing primer was then annealed to the SMRTbell template, followed by binding of sequence polymerase to the annealed template. Finally, one SMRT cell was run on the PacBio PacBio Sequel System to sequence the sample.

In addition, whole genome shotgun (WGS) sequencing approach was performed on both H2 and H3 isolates. 2 µg of gDNA of each isolate were fragmented and size selected as described above. Paired-end short read sequencing libraries (Illumina HiSeq 2500 System, 2 × 100 bp) were prepared with Illumina HiSeq Rapid v2 SBS Kits. The short-read libraries were sequenced on an Illumina HiSeq 2500 System, per standard Illumina protocols (Illumina, Inc., San Diego, CA, United States). The sequencing adapters in the PacBio CLR reads and the 2 × 100 Illumina HiSeq reads were removed by the sequencing company.

### Sequence data processing, genome assembly, gene prediction and genome annotation

The genome size estimation was performed using the k-mer analysis of the 2 × 100 Illumina HiSeq reads from the H3 isolate using the k-mer of sizes 17, 19, and 21 under the Jellyfish software (Marçais and Kingsford 2011). The frequency of the k-mers and the depth were plotted for the k-mer frequency distribution. An error threshold of four was used to eliminate the noise peak likely due to sequencing errors. Genome size was estimated by dividing the total number of k-mers by the peak position. The genome coverage for Illumina was calculated by dividing the total number of reads by the genome size estimate with the different k-mer sizes. The genome coverage was also estimated from the assembled genome of *C*. aff. *paspali* H3.

The genome assembly of the *C*. aff. *paspali* was performed using PacBio reads of isolate H3 under the fork of the canu version 2.2 (Koren et al., 2017). Default parameters were used with an estimated genome size of 42 MB (Conservative genome estimate taken from the k-mer analysis of Jellyfish). The assemblies were polished using the Illumina reads (∼35X read coverage) of isolate H3 under Pilon using the settings “--fix all--mindepth 0.5.” The resulting contigs were BLAST to the NCBI “nr” database to contaminants. An e-value cutoff of 1e-10 was used. Contigs (74 from 194 total contigs) which had matches to the “nr” database at the stringent cut-off were manually inspected for the presence of non-plant contaminations. One contig (tig00001304_pilon; size = 2200 bp) showed homology to a herpes virus genome (e-value = 0) over its entire length and was removed from downstream analyses. The H2 genome assembly was performed using SPAdes version 3.15 (Bankevich et al., 2012). The genome coverage was estimated for the final genomes of the H2 and H3 isolates. The completeness of the genome of *C*. aff. *paspali* (H3 isolate) was assessed by the Benchmarking universal Single Copy Orthologs (BUSCO) (Simão et al., 2015) compared to a reference dataset of fungi (“fungi_odb10”) and eukaryote (“eukaryote_odb10”). Published genomes of *C. jacksonii* (LWC-10 isolate, Crouch et al., 2021), *C. monteithiana* (DRR09 isolate; Crouch et al., 2021) and *S. sclerotiorum* (1980 UF-70 isolate; GenBank Acc. No. GCA_001857865.1) were added for the comparative analysis. In addition, RepeatMasker v4.0.9 (http://www.repeatmasker.org/) was used to assess the repetitive content in the genome using the settings “-parallel 40 -html -gff -e rmblast -e ncbi.”

The soft masked genome generated from the RepeatMasker was used to for the gene prediction. The *ab initio* gene prediction was performed under AUGUSTUS v3.4 using the species *Sclerotinia sclerotiorum* genome (1980 UF-70 isolate; GenBank acc. no GCA_001857865.1) and using the hints file generated from the BLAST analysis of the EST sequences from *C. monteithiana* (DRR09; Crouch et al., 2021) and *C. jacksonii* (MB01; Crouch et al., 2021). The aligned ESTs were filtered for a minimum coverage of 80% (filterPSL.pl--best--minCover = 80) and the hint file was generated. Furthermore, the functional annotation prediction of the *C*. aff. *paspali* (H3) was performed using the INTERPRO scan (Blum et al., 2021) under default settings. We used the setting “--goterms--pathways--iprlookup” in INTERPRO scan that assigned the GO term to the genes. The top 10 occurrences of the GO terms were identified from this INTERPRO list for cellular component, biological process and molecular function.

### Ortholog analysis and genomic similarity

The similarity in the nucleotides at the whole genome level among the *Clarireedia* species was performed to understand how they are related to each other. Two isolates for each species were used: *C. jacksonii*, *C. monteithiana* and *C*. aff. *paspali* were represented by LWC-10 (Crouch et al., 2021) and HRI11(Green et al., 2016), DRR09 and RB-19 (Crouch et al., 2021), H3 and H2 isolates, respectively. The *Clarireedia* genomes were also compared with *S. sclerotiorum* (1980 UF-70 isolate; GenBank acc. no GCA_001857865.1) and *R. sydowiana* (CBS115975 isolate; GenBank acc. no JWJB00000000). For this analysis, the program FASTAni (Jain et al., 2018) was used with default settings. Furthermore, the different *Clarireedia* species and *S. sclerotiorum* were compared on the orthologous genes. Identification of orthologous genes in *C*. aff. *paspali* (H3) was performed using the protein models from AUGUSTUS under Orthofinder (Emms and Kelly 2019) with default settings with published gene models of *C. jacksonii* (LWC-10), *C. monteithiana* (DRR09) and *S. sclerotiorum* (1980 UF-70 isolate). An orthogroup can comprise one or more gene models from at least one species. The overlap between ortho groups across species was plotted using the Upset plot (https://asntech.shinyapps.io/intervene/). Because no gene models were available for *R. sydowiana*, the comparison of orthogroups was not possible.

### Molecular biology of morphogenesis and pathogenesis, and phylogeny

The molecular biology of *C*. aff. *paspali* morphogenesis and pathogenesis was investigated at the genomic and protein levels. We used the 13,428 protein models identified through AUGUSTUS and ran it through the EffectorP version 3 (Sperschneider and Dodds, 2022) using the default settings. The program identified weather the gene model is either a cytoplasmic effector, apoplastic effector, both or a non-effector. We used a cut-off of *p*-value > 0.9 to designate the cytoplasmic and apoplastic effectors. A BLASTP analysis to the protein database of NCBI was performed on these effectors and the top 10 hits were noted.

#### At the genomic level

The genome of *C*. aff. *paspali* was compared with *C. monteithiana* and *C. jacksonii* as well as *S. sclerotiorum* (1980 UF-70 isolate), as a representative of the Sclerotiniaceae family, for the presence/absence of a range of fifty-four possible determinants involved in pathogen morphogenesis and pathogenesis (Supplementary Table S1). These determinants were previously characterized in *S. sclerotiorum* (Xia et al., 2019), the closest fully sequenced and well characterized relative species to *Clarireedia*. LWC-10 and HRI11 (Green et al., 2016; Crouch et al., 2021), DRR09 and RB-19 (Crouch et al., 2021), H3 and H2 isolates, representative of *C. jacksonii*, *C. monteithiana*, and *C*. aff. *paspali* were used in this analysis. For each gene present in *Clarireedia*, the average percentage of similarity between *Clarireedia* spp. and *S. sclerotiorum* at the DNA and protein levels were also recorded using NCBI blast. Species-specific SNP variations as well as non-synonymous and non-conservative SNPs between the *Clarireedia* species within these pathogenicity genes was also investigated for the genes that were present in all three species *C. aff. paspali*, *C. monteithiana*, and *C. jacksonii*. Species-specific SNPs are SNPs present in both isolates of a particular *Clarireedia* species and absent in isolates of the other two species. Non-conservative SNPs are non-synonymous substitutions that change the property of the corresponding amino acids. This comparative analysis was also performed on five conserved genomic sequences of the ITS, TUB, CaM, EF and Mcm7 regions. BLAST analysis of the curated pathogenicity and conserved genes was performed using the default settings to extract the sequences from the different assembled genomes. The *S. sclerotiorum* cDNA sequences were used as templates to assign the start and the end of the gene. We also included in this comparative analysis the sequences from the plant pathogen *R. sydowiana* (CBS115975 isolate) as a representative of the Rutstroemiaceae family, and the sequences from the plant pathogen *Z. tritici* (CBS115943 isolate; https://fungi.ensembl.org/Zymoseptoria_tritici/Info/Index) causal agent of septoria leaf blotch as an outgroup.

#### At the protein level

The nucleotide sequences of the 13 virulence genes (present across the Clarireedia species) from *S. sclerotiorum* were translated using MEGA X (Kumar et al., 2018) software, and the peptide sequences obtained were used to predict the three-dimensional structure of the selected virulence genes using a web-based protein structure prediction tool, Protein Homology/analogy Recognition Engine (Phyre2) version 2.0 (Kelley et al., 2015). The predicted structures having coverage >80% were selected, followed by prediction of structures of the respective virulence genes from *C. jacksonii*, *C. monteithiana*, and *C.* aff. *paspali*. The molecular graphics, structural alignment, and comparison of the proteins from *S. sclerotiorum* and the three *Clarireedia* spp. was performed using the MatchMaker extension of UCSF Chimera 1.15 (Pettersen et al., 2004). Along with constructing the molecular graphics of the predicted models, movie animation of the aligned models was created to distinguish the structural differences using UCSF Chimera 1.15. In addition, the quality of the structures predicted was validated using two model validation online tools, the Structure Analysis and Verification Server (SAVES) v6.0 and Qualitative Model Energy ANalysis (QMEAN) (Benkert et al., 2011). The different programs in SAVES v5.0 such as PROCHECK (Laskowski et al., 1996), VERIFY-3D (Bowie et al., 1991; Lüthy et al., 1992) and ERRAT (Colovos and Yeates 1993), were used to provide the stereochemical attributes of the structure in the form of Ramachandran plot, atomic-resolution coordinates of the 3D models, and comprehensive quality factor of the non-bonded atoms in the structure, respectively. The quality score of the model was also evaluated as Z-scores using QMEAN tool.

### Phylogenetic analysis

The extracted sequences of six pathogenicity genes (*shk1*, *ste12*, *smk3*, *pph1*, *gpd*,* nox2* that were present in all three *Clarireedia* species as well as in *R. sydowiana*) and the five conserved genes (ITS, TUB, CaM, EF and Mcm7) were concatenated into one contiguous sequence for each isolate, aligned with MUSCLE (Edgar 2004) and used for phylogenetic analysis. A neighbour joining (NJ) tree based on the maximum composite likelihood method was performed under Mega X (Kumar et al., 2018) with a 500 replicates bootstrap test. In addition, divergence-time estimates within *Clarireedia* species and between *Clarireedia* and *S. sclerotiorum* were also calculated using an average relative divergence time of 437 million years ago (Mya) between *S. sclerotiorum* and *Z. tritici* as standard (www.timetree.org). Furthermore, a molecular clock test was conducted in MEGA X (Kumar et al., 2018) and was performed by comparing the Maximum Likelihood value for the NJ topology with and without the molecular clock constraints under Tamura-Nei model (Tajima 1993). The analysis was performed on a total of 22,983 positions of the nine nucleotide sequences combining the virulence and the conserved gene regions. The nine nucleotide sequences were from the *Clarireedia* isolates LWC-10 and HRI11 (Green et al., 2016; Crouch et al., 2021), DRR09 and RB-19 (Crouch et al., 2021), H3 and H2, as well as from *S. sclerotiorum* (1980 UF-70 isolate), *Rutstroemia sydowiana* (CBS115975 isolate), *Zymoseptoria tritici* (CBS115943 isolate). In addition, the Tajima’s relative rate test (Tamura and Nei 1993) was performed under MEGA X (Kumar et al., 2018) between *C.* aff. *paspali* (H3), *C. jacksonii* (LWC-10) and *C. monteithiana* (DRR09), with *Z. tritici* (CBS115943) sequence as an outgroup.

## Results

### Sample identification and pathogenicity

**FIGURE 1:**
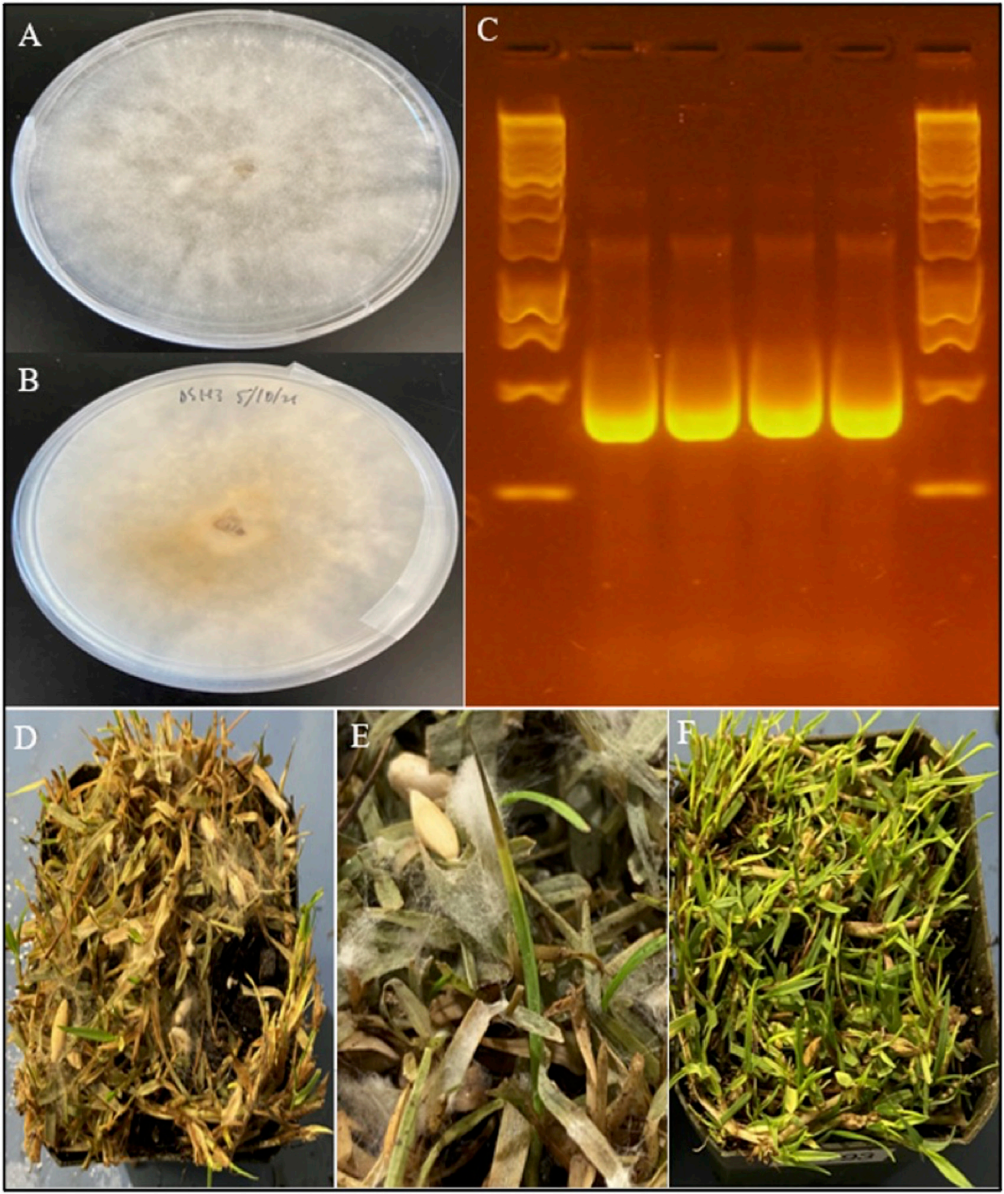
Morphology, molecular identification, and symptoms of *C.* aff. *paspali*. **(A)** Front and **(B)** back of a 5-day old *C*. aff. *paspali* culture (H3 isolate) on PDA media. **(C)** Molecular characterization of H2 (no. 1) and H3 isolates (no. 2) of *C*. aff. *paspali* along with two reference isolates DS-SP of *C. monteithiana* (no. 3) and DS-CB of *C. jacksonii* (no. 4) on the ITS region (from left to right) showing no differences in band sizes between *Clarireedia* species. A Thermo Scientific GeneRuler 1 kb DNA Ladder was used. **(D)** Pathogenicity tests showing signs and symptoms of *C.* aff. *paspali* infection and **(E)** foliar tip dieback and aerial mycelia growth. **(F)** Non-inoculated control.

Pure cultures of H2 and H3 presented an aspect with a white fluffy mycelium on PDA media, typical for dollar spot (Figures 1A,B). From the pathogenicity tests, aerial mycelial growth from both H2 and H3 inoculations, as well as for the DS-SP *C. monteithiana* isolate, was observed in all four turfgrass species 2 days after inoculation. Dollar spot foliar symptoms (lesions, blight, tip dieback) caused by H2, H3 and DS-SP infection were prominent in all turfgrass species 2 weeks after inoculation (Figures 1D–F). Symptoms were most severe on seashore paspalum. No differences in aggressiveness were observed between the 2 *C.* aff. *paspali* isolates and DS-SP *C. monteithiana* isolate when inoculated on the four different turfgrass species. Control plants showed no symptoms. The pathogen was re-isolated from the artificially inoculated plants. Results of the pathogenicity tests indicate that both H2 and H3 isolates were able to infect cool- and warm-season turfgrass species under favorable conditions. In addition, preliminary tests showed that H3 *C.* aff *paspali* isolate did not show any differences in aggressiveness compared to DS9 and DS7 *C. monteithiana* isolates (sampled at Griffin, GA in 2019 on berdumagrass and zoysiagrass, respectively) and DS-CB *C. jasksonii* isolate (sampled from Griffin, GA in 2019 on creeping bentgrass) when inoculated on seashore paspalum cv. SeaStar, bermudagrass cv. Princess, zoysiagrass cv. Zorro and creeping bentgrass cv. A-1/A-4 (data not shown). However, additional investigations need to be carried out at the population level to compare the aggressiveness of this new species *C.* aff. *paspali* to the previously described species in the United States.

BLAST results against the NCBI database of H2 and H3 Sanger sequences exhibited 98.07%–99.37% sequence homology to *C. homeocarpa* (GenBank acc. no. KF545301.1, MF969128.1, DQ448301.1, KF545258.1 for ITS, TUB, EF and CaM respectively). Based on sequence comparison with *Clarireedia* reference isolates from Hu et al. (2019), H2 and H3 isolates showed the highest homology (100–99.59%) with isolate HK1 of *C.* aff. *paspali* on the ITS and EF regions, and did not present the intron at the 3′-end of the SSU rDNA region, characteristic of *C. paspali* (Figure 1C). In addition, H2 and H3 did not carry any of the SNPs specific to *C. jacksonii*, *C. monteithiana*, *C. homeocarpa* or *C. bennetti* identified in the ITS region by Salgado-Salazar et al. (2018). However, H2 and H3 isolates presented 1 (“C” at position 131; Salgado-Salazar et al. (2018)) and 2 (“C” at position 64 and “C” at position 483; Salgado-Salazar et al. (2018)) species-specific SNPs for *C.* aff. *paspali* in the ITS and EF genes, respectively.

### Genome assembly of *C.* aff. *paspali*

Draft genome sequence data, generated using an Illumina 550 bp PE-SRS library, generated 14,99,240,400 of total raw reads (H2 and H3 isolate) and 14,73,271,400 and 14,90,276,400 of total high-quality reads for the H2 and H3 isolates, respectively. The H3 isolate was sequenced with the long-read PacBio sequencing platform with a total of 163,709 sequenced reads for a total of 13.90 gigabases.

**FIGURE 2:**
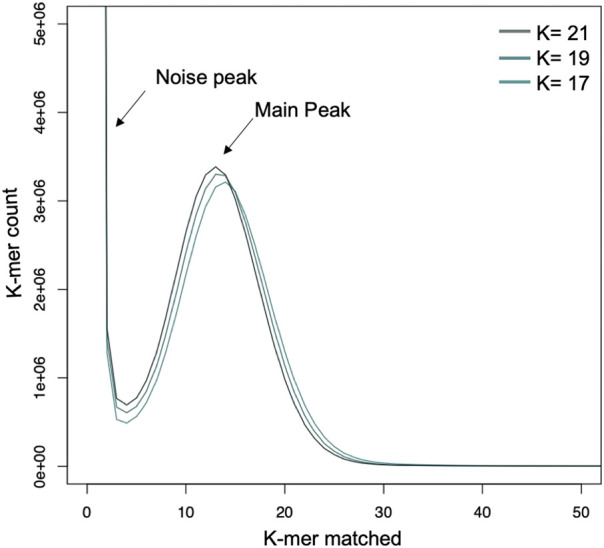
Genome estimation using k-mer analysis with 17-, 19- and 21- mers. The k-mers show a peak at a similar location which gives an estimate of 35 X genome coverage and a genome size of 48 MB for *C*. aff. *paspali* (H3 isolate).

The main peak in the k-mer frequency distribution graph gives a matched k-mer value for the genome size estimation. Noise peaks were formed by very low coverage error k-mers (noise; below *k* = 4) from sequencing errors. The highest peak was observed at estimated depths of 35X, 34X, and 35X for the 17-, 19-, and 21-mers, respectively (Figure 2). The presence of a single dominant peak indicates that the genome is only slightly repetitive and haploid. According to the k-mer size estimations, the estimated genome size of *C.* aff. *paspali* H3 isolate ranged between 42–44 Mb with ∼316X of coverage (with 44 Mb genome size).

The error corrected PacBio + HiSeq draft assembly of *C.* aff. *paspali* H3 isolate contained a total of 193 contigs with ∼48.6 Mbp and contig N50 length was 5 Mb (Table 1). The top 23 contigs represented >90% of the total genome (43.8 Mbp). The sizes of the two largest contigs for *C*. aff. *paspali* H3, tig00000001 and tig00002064, were 10.2 Mbp and 5.9 Mbp, respectively (Supplementary Table S2). The *de novo* assembly for *C.* aff. *paspali* H2 isolate produced 6,743 contigs with 39.26 Mbp, and ∼77 kb contig N50 with HiSeq sequencing method. Based on a 48.6 Mb estimate, we generated more than ∼286X coverage (size of genome/total # of bases) of the H3 *C.* aff. *paspali* genome using one SMRT cell. The H2 isolate was assembled using Illumina reads with ∼37.53 X coverage of the genome.

**TABLE 1:** Sequencing summary statistics of *C.* aff. *paspali* genome (H3 isolate).

A. Sequencing reads	Illumina HiSeq PE	PacBio
Raw Reads	14,99,240,400	
Processed Reads	14,90,276,400	163,709 (13.90 Giga bases)
B. Assembly data		
No. of Contigs	193 (48.594 Mb)^a^	
Average length	251784.3 bp	
Min. Length	441	
Max. Length	10250504	
N50	5 Mb	
N%	--	
GC%	38.53%	
C. Genetic Elements		
Number of Genes	13,428	
Avg. gene length	1218.38 bp	
Avg. exon length	545.92 bp	
Repeat Elements	2.23%	
Genome Coverage	32.32%	
D. Annotation		
BLAST hits (%)^b^	34.77	
No Hits (%)	65.23	
GO annotated (%)	47.86	
E. Top 10 GOs^c^		
Cellular Component	Biological Process	Molecular Function
Integral component of membrane (362)	Transmembrane transport (529)	Protein Binding (733)
Membrane (323)	Regulation of transcription, DNA-templated (325)	ATP Binding (537)
Nucleus (122)	Carbohydrate metabolic process (270)	Oxidoreductase activity (419)
Ribosome (105)	Transcription, DNA Templated	Zinc Ion Binding (396)
Cytoplasm (90)	Proteolysis (140)	Transmembrane transporter activity (371)
Extracellular region (50)	Protein phosphorylation (126)	Catalytic Activity (289)
Mitochondrion (22)	Translation (118)	DNA Binding (268)
Mediator Complex (21)	DNA repair (73)	Nucleic Acid Binding (250)
Mitochondrial Inner Membrane (17)	Lipid Metabolic Process (56)	DNA binding transcription factor activity, RNA polymerase II specific (232)
Proteosome core complex (15)	Mycotoxin Biosynthesis Process (54)	RNA Binding (180)

^a^ Assembly was performed using the PacBio reads and errors were corrected using Illumina reads.

^b^ BLAST analysis was performed on the predicted genes to the NCBI nucleotide “nt” database.

^c^ The number between backets is the occurrence of the GO term.

We did not want to mask the low complexity repeat elements as some of these repeat elements are present within the coding regions. The un-masked genome was used as input in Augustus.

### Repeat analysis, gene prediction, and gene ontology annotation

**FIGURE 3:**
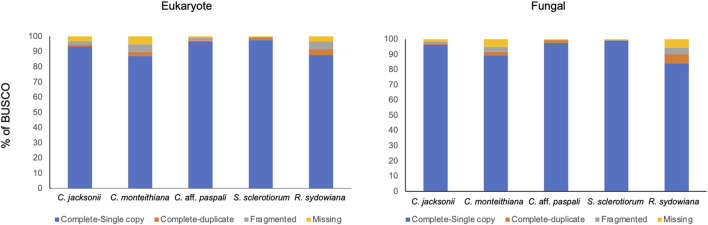
Benchmarking Universal Single Copy Orthologs (BUSCO) analysis of gene models using the eukaryote obd10 (*n* = 255; species = 70) and the fungal obd10 (*n* = 758; species = 549) BUSCOs. Gene models generated in this paper from the Augustus pipeline were used for the *C*. aff. *paspali* (H3 isolate). Published gene models were used for *C. jacksonii* (LWC-10), *C. monteithiana* (DRR09), and *R. sydowiana* (CBS115975), and *S. sclerotiorum* (1980 UF-70).

We submitted ∼48.6 Mbp of the *C.* aff. *paspali* genome to RepeatMasker and observed that only 2.23% of the genome was classified as repetitive, indicating a low complexity genome. The majority (1.82%) of the repeat elements were the simple repeats (microsatellites, simple sequence repeats, or short tandem repeats) and a smaller percentage were low-complexity repeats (0.28%). Other repeat elements included LINEs (0.04%), DNA repeats (0.01%), total interspread repeats (0.05%) and small RNA (0.09%). No homology to DNA transposons and retrotransposons was noted in the RepeatMasker analyses. According to our RepeatMasker analysis, *C. jacksonii*, *C. monteithiana*, *S. sclerotiorum*, and *R. sydowiana*, presented similarly low repetitive genomes with 2.13%, 2.02%, 2.47%, and 1.89% of repeats, respectively. To assess the completion of the genome, we performed a BUSCO analysis. The genome of the *C.* aff. *paspali* H3 isolate comprised 99.3% of the complete BUSCOs (97.6% single copy, 1.7% duplicated copy) using the fungal database. In addition, 0% of the gene models were fragmented BUSCOs and 0.7% were missing (Figure 3). In comparison to other *Clarireedia* species, *C.* aff. *paspali* showed the highest number of Complete BUSCOs and similar statistics were also observed with the EUKARYOTIC BUSCO dataset (Figure 3).

Using the trained *de novo* prediction from AUGUSTUS, we predicted 13,428 gene models (18.406 MB), with an average gene length of 1,360.7 bp, which constitutes 37.88% the *C.* aff. *paspali* genome (Table 1). We also identified that 34.77% (4,669 genes out of 13,428) of the discovered annotated gene models contain BLAST hits to the NCBI “nt” database. From the Interpro scan analysis, we assigned GO categories to 6,247 genes (47.86% of the total genes). Table 1 provides the top 10 GO terms in each of the categories of GO. The top GO term in cellular component, biological process and molecular function are Integral component of membrane (*n* = 362), Transmembrane transport (*n* = 529) and Protein Binding (*n* = 733) respectively.

### Ortholog analysis and genomic similarity

**FIGURE 4:**
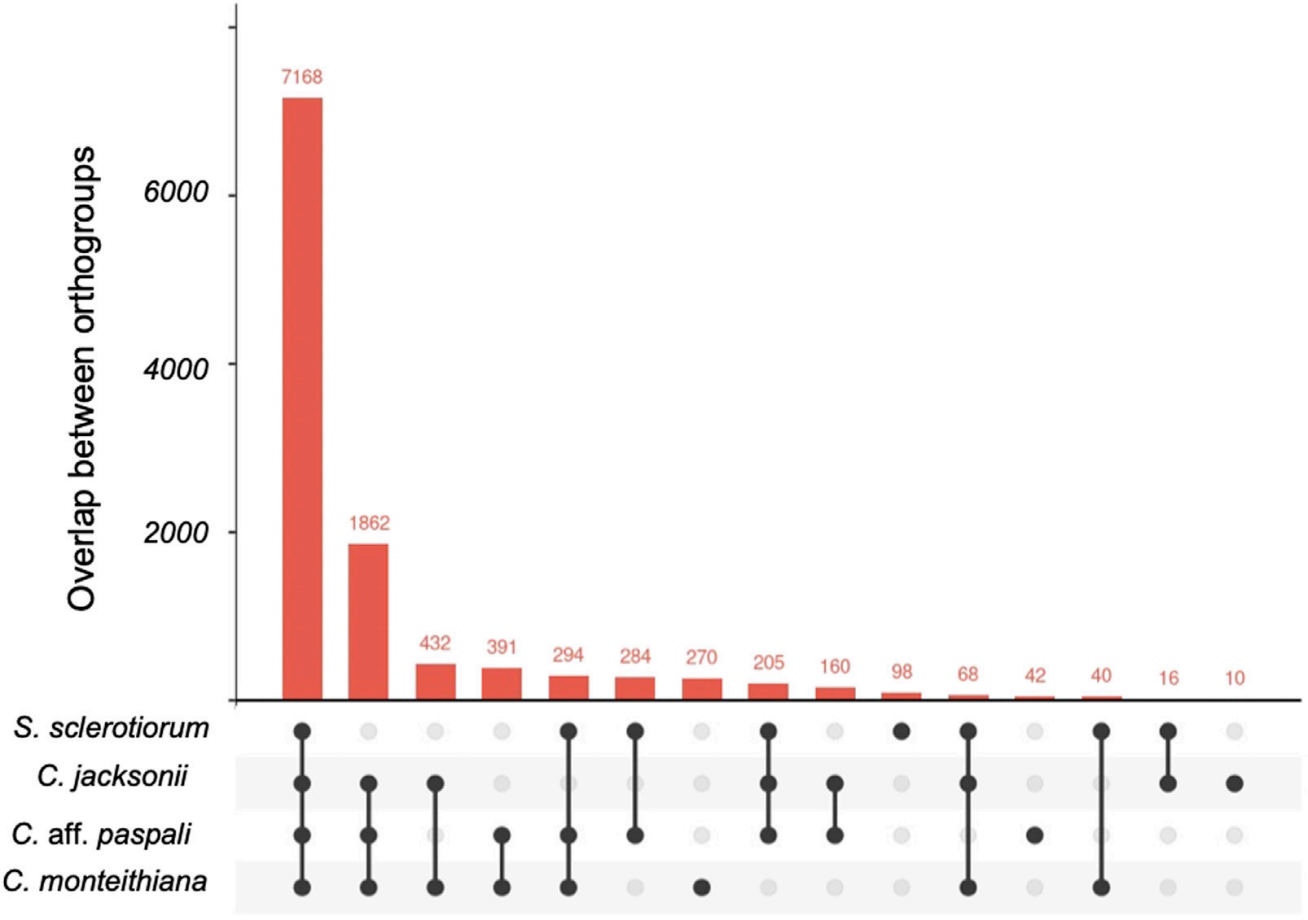
Upset plot of orthologous gene clusters between *C.* aff. *paspali* and other related species. One to several genes can be present within each ortho gene cluster. *S. sclerotiorum*, *C. jacksonii*, *C. monteithiana*, and *C*. aff. *paspali* are represented by the genome of isolates 1980 UF-70, LWC-10, DRR09, and H3, respectively.

A total of 11,340 ortho-clusters were identified in the analysis of the three *Clarireedia* species and *S. sclerotiorum* (Figure 4). The ortholog analysis revealed 10,580 orthologous clusters containing at least two *Clarireedia* species, which constitute 93.3% of the total orthologous clusters. In addition, 322 unique *Clarireedia* species-specific orthologous groups and 1,862 orthologous groups common to all the studied *Clarireedia* species, were identified. Interestingly, there were more orthogroups common between *C.* aff. *paspali* and *C. monteithiana* (*n* = 391) than *C.* aff. *paspali* and *C. jacksonii* (*n* = 160). Interestingly, at a pairwise level, *C. jacksonii* and *C. monteithiana* (*n* = 432) had the highest orthogroups in common. Within *Clarireedia*, *C. monteithiana* had the highest number of species-specific orthologous groups (270 orthologous groups), followed by *C.* aff. *paspali* (42) and *C. jacksonii* (10) (Figure 4).

**FIGURE 5:**
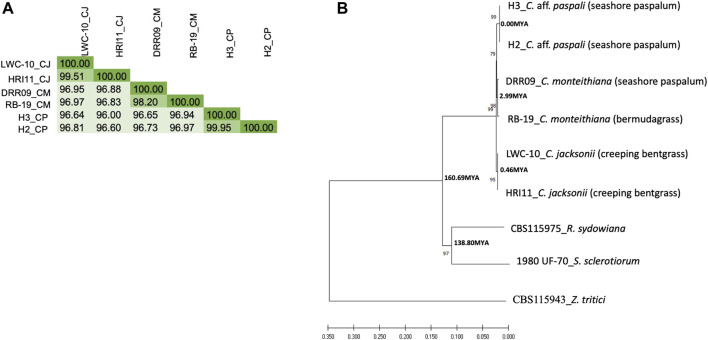
Genetic relationship of *C.* aff. *paspali* with related species. **(A)** DNA similarity matrix between the *Clarireedia* species. *C. jacksonii* (CJ), *C. monteithiana* (CM) and *C.* aff. *paspali* (CP) were represented by LWC-10 and HRI11, DRR09 and RB-19, H3 and H2 isolates, respectively. **(B)** Neighbor-joining tree of *Clarireedia* spp. and related species conducted from combined virulence and conserved gene sequences. Numbers associated with branches are bootstrap percentage. Two isolates for each *Clarireedia* species were used. *S. sclerotiorum* (1980 UF-70) and *R. sydowiana* (CBS115975) were used as representatives of the Sclerotiniaceae family and the Rutstroemiaceae family, respectively. *Z. tritici* (CBS115943) was used as an outgroup. Numbers associated with nodes are estimated divergence times. When known, the host origin of the isolate was indicated between brackets.

From the FastANI analysis of genome similarity, we observed, as expected, more similarity within *Clarireedia* species (>98%) than between *Clarireedia* species (≤96%) (Figure 5A). 99.51% of sequence similarity within *C.* aff. *paspali* (between H2 and H3 isolates) was observed. Within species analysis showed the lowest genome similarity between the two isolates of *C. monteihiana* DRR09 and RB-19. On average, *C.* aff. *paspali* (H2 and H3) showed more genome similarity with *C. monteihiana* (96.82%) isolates (DRR09 and RB-19) than with *C. jacksonii* (96.51%) isolates (LWC-10 and HRI11). The genome similarity values of *S. sclerotiorum* and *R. sydowiana* with the *Clarireedia* species could not be identified as the similarity dropped below 80% and the program could not assign a value.

### EffectorP analysis

Out of the 13,428 protein models, we identified 357 cytoplasmic effectors and 13 apoplastic effectors (with *p*-values > 0.9) which in combine constitute 2.68% of the total identified protein models. 349 cytoplasmic effectors and 13 apoplastic effectors had BLAST hits to the NCBI “nr” protein database (Supplementary Table S3). Sixteen and 136 cytoplasmic effectors blasted against *Sclerotinia* spp. and *Rutstroemia* spp., respectively. In addition, five apoplastic effectors blasted against *Rutstroemia* spp., while none blasted against *Sclerotinia* spp. Three hundred and forty-nine cytoplasmic effectors and 13 apoplastic effectors were identified in 33 contigs of the *C.* aff. *paspali* genome; two major contigs (tig00000001 and tig00002064) grouped 36% of the effectors. The average densities were four cytoplasmic effectors every 100 kb and nine apoplastic effectors every 10 Mb. Three contigs (tig00000377, tig00000363, and tig00000354) harbored surprisingly a higher effector density (three cytoplasmic effectors every 10 kb) (Supplementary Table S3).

### Molecular biology of *C.* aff. *paspali* morphogenesis and pathogenesis

We screened the *C.* aff. *paspali* genome and related species for the fifty-four biological determinants compiled by Xia et al. (2019) involved in *S. sclerotiorum* morphogenesis and pathogenesis. Only seventeen genes (∼31% of the biological determinants) were present in at least 1 *Clarireedia* species (Table 2; Supplementary Table S1). Out of these seventeen pathogenesis genes, four were predicted by EffectorP as cytoplasmic effectors (*caf1*, *pka1*, *nacα*, and *gpd* genes) with *p*-values of 0.7-0-8 (Supplementary Table S1). Seven genes (*cna1*, *caf1*, *nox1*, *pac1*, *pka1*, *ams2*, and *nacα)* were present in all the *Clarireedia* species but absent in the sister genus *R. sydowiana*; however, three genes (*pth2*, *bi1*, and *hex1*) are absent in all the *Clarireedia* species but present in *R. sydowiana*. Four genes showed presence/absence variation between the *Clarireedia* species. *rgb1* and *rhs1*, genes involved in virulence, appressoria formation and sclerotial formation, were exclusively present in both isolates of *C. monteithiana* and *C.* aff. *paspali*, respectively. Adenylate cyclase (*sac1*), a gene involved in virulence, hyphal growth and sclerotial formation was absent in both isolates of *C.* aff. *paspali*. Another gene, *pks13*, a polyketide synthase, involved in appresoria formation, was absent in both *C.* aff. *paspali* isolates and in 1 *C. monteithiana* isolate (RB-19 isolate sampled from bermudagrass). SNP variations among the genes were assessed in the thirteen virulence genes present across the *Clarireedia* species. A total of 366 species-specific SNPs were recorded. *C. jacksonii* (151 SNPs) and *C. monteithiana* (78 SNPs) presented the highest and lowest number of species-specific SNPs, respectively. The gene *ste12*, coding for downstream transcription factor of MAPK pathway, contained the highest number of species-specific SNPs for *C.* aff. *paspali* (25 SNPs) and for *C. jacksonii* (26 SNPs). However, the highest number of species-specific SNPs (11 SNPs) for *C. monteithiana* was observed in *ams2* gene (GATA transcription factor; 11 SNPs) and caf1 gene (compound appressorium formation related gene 1; 10 SNPs). Of the 366 species-specific SNPs, only 28 SNPs were non-synonymous and non-conservative (7.6%). Overall, the percentage of protein similarity of *S. sclerotiorum* with *Clarireedia* species ranged from 58.39% (CNA1 protein) to 96.52% (AMS2 protein) with an average of 86.07%. NOX1 and NOX2 showed no polymorphism between *Clarireedia* species at the protein level and a high percentage of protein identity with *S. sclerotiorum* (91.76%–93.58%).

**TABLE 2:** Summary of *S. sclerotiorum* orthologs involved in pathogen morphogenesis and pathogenesis identified in *C.* aff. *paspali* and their genetic variation between *Clarireedia* species.

*S. sclerotiorum* virulence genes	Mutant Name^a^	Gene full name^a^	Gene size (bp)^c^	*C.* aff. *paspali*	*C. monteithiana*	*C. jacksonii*	*R. sydowiana*
sscle_01g006030	*cna1*	Catalytic subunit calcineurin-encoding gene	1807 (81.75/96.52)	+(6)	+(5:2)	+(8)	-
sscle_02g013550	*shk1*	Histidine kinases	4321 (81.81/75.98)	+(13:2)	+(4:1)	+(14:4)	+
sscle_03g031520	*pks13*	Polyketide synthase		-	+/-^b^	+	+
sscle_03g031670	*pth2*	Peroxysomal carnitine acetyl transferase		-	-	-	+
sscle_04g034960	*caf1* ^d^	Secreted protein with a putative Ca2+-binding EF-hand motif	773 (76.71/82.83)	+(8:1)	+(10)	+(13)	-
sscle_05g046790	*bi1*	Bax inhibitor-1 protein		-	-	-	+
sscle_05g048220	*nox1*	NADPH oxidase	1685 (79.98/91.76)	+(9)	+(7)	+(11)	-
sscle_06g049430	*rhs1*	Rearrangement hot spot repeat-containing protein		+	-	-	-
sscle_06g049830	*pac1*	pH-Responsive transcription factor	2068 (73.51/75.99)	+(8)	+(4:1)	+(16:5)	-
sscle_06g051560	*ste12*	Downstream transcription factor of MAPK pathway	2379 (79.33/90.89)	+(25:6)	+(8)	+(26)	+
sscle_07g055970	*hex1*	Woronin body major protein		-	-	-	+
sscle_07g058030	*ams2*	Cell-cycle-regulated GATA transcription factor	2253 (84.81/58.39)	+(24:4)	+(11:1)	+(13)	-
sscle_07g058620	*pka1* ^d^	Protein kinase A	1548 (78.47/89.15)	+(3)	+(5)	+(9)	-
sscle_08g065550	*nacα* ^d^	Nascent polypeptide-associated complex α-subunit	850 (82.85/87.56)	+(5)	+(1)	+(8)	-
sscle_08g066770	*smk3*	Slt2 ortholog	1488 (79.69/90.75)	+(11)	+(5)	+(5)	+
sscle_10g077630	*pph1*	Type 2A Ser/Thr phosphatase catalytic subunit PP2Ac	1254 (79.74/94.62)	+(6)	+(3)	+(15)	+
sscle_11g082700	*rgb1*	Type 2A Ser/Thr phosphatase B subunit		-	+	-	+
sscle_11g083230	*gpd* ^d^	glyceraldehyde-3-phosphate dehydrogenase	1223 (83.51/90.94)	+(10:1)	+(7)	+(7)	+
sscle_11g083950	*sac1*	Adenylate cyclase		-	+	+	-
sscle_12g087830	*nox2*	NADPH oxidase	1871 (81.98/93.58)	+(9)	+(8)	+(6)	+

“+” indicates the presence of the BLAST hit to the genome and “-” indicates absence. The first number between brackets indicates the number of species-specific SNPs that are present in both isolates of the corresponding *Clarireedia* species and absent in all the other four isolates of the other *Clarireedia* species. The second number between brackets indicates the number of species-specific SNPs that are non-synonymous and non-conservative. *C*. aff. *paspali* is represented by H2 and H3 isolates; *C. monteithiana* is represented by H2 and H3 isolates; *C. jacksonii* is represented by LWC-10 and HRI11 isolates; *S. sclerotiorum* is represented by UF-70isolate; *R. sydowiana* represented by CBS115975 isolate.

^a^ Column subsets have been taken from Table 1 from Xia et al., 2019.

^b^ Pks13 is present in DRR09 isolate and absent in RB-19 isolate.

^c^ Average percentage of similarity between *S. sclerotiorum* and *Clarireedia* spp. at the DNA level followed by the protein level are given between brackets.

^d^ Indicates pathogenesis genes that were predicted by EffectorP as cytoplasmic effectors in *C.* aff. *paspali*.

### Protein structure prediction, visualization, and evolution

**FIGURE 6:**
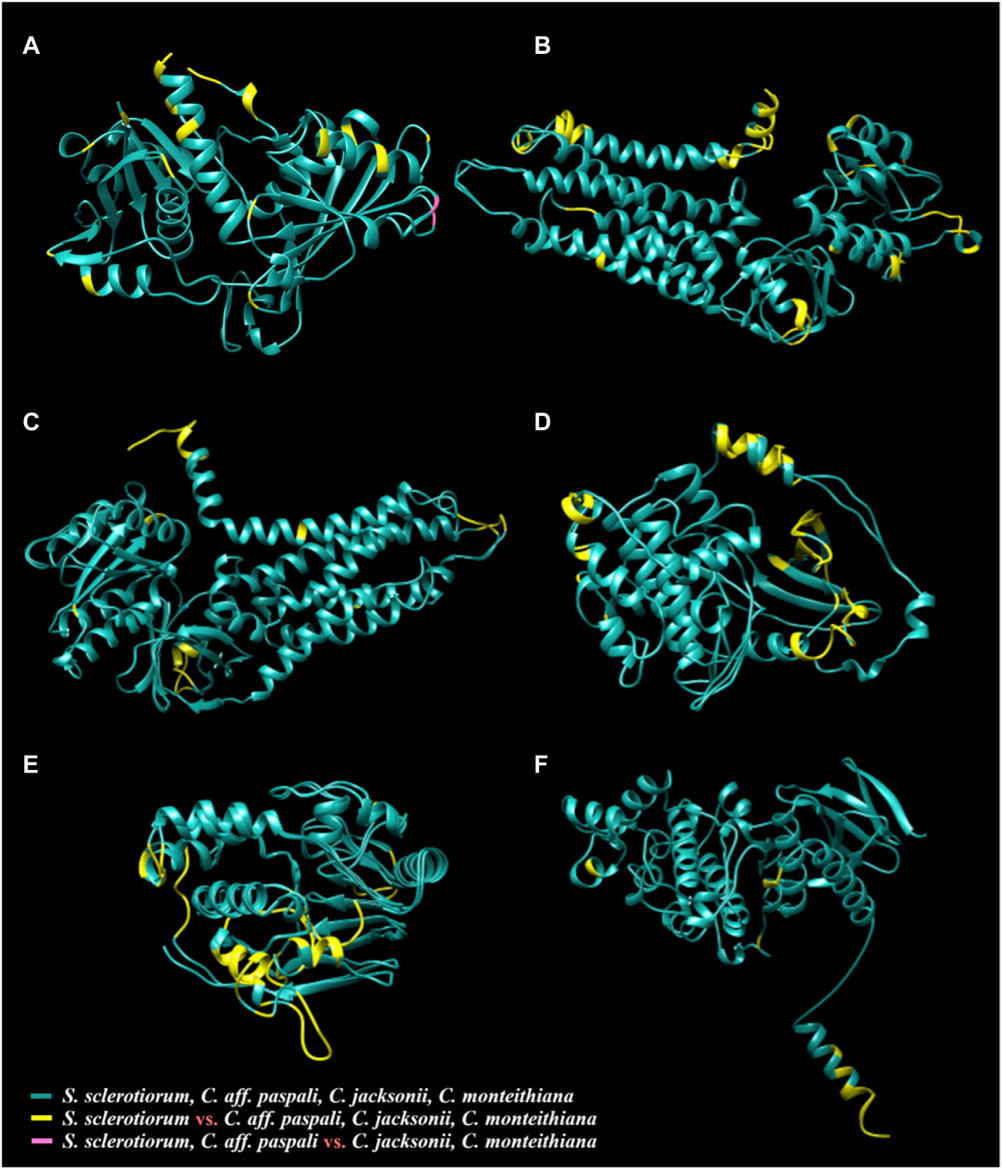
Three-dimensional protein models of six virulence genes [**(A)** *gpd*, **(B)** *nox1*, **(C)** *nox2*, **(D)** *pka1*, **(E)** *pph1*, and **(F)** *smk3*] from *S. sclerotiorum* aligned with the respective protein structures from *C. jacksonii*, *C. monteithiana*, and *C.* aff. *paspali*. The common structures between all species are in green. The species-specific structures of *S. sclerotiorum* that are distinct from all *Clarireedia* (*C. jacksonii*, *C. monteithiana* and *C*. aff. *paspali*) are highlighted in yellow. The single region in GPD (A) protein differentiating *C*. aff. *paspli* from *C. jacksonii* and *C. monteithiana* is color-coded in pink.

Protein modelling of the thirteen virulence genes of *S. sclerotiorum* was performed, and the models which were predicted with high coverage (more than 80%) were selected to assess the alignment of the respective proteins from *Clarireedia* spp. (Supplementary Table S4). Six models (GPD, NOX1, NOX2, PKA1, PPH1, and SMK3) from *S. sclerotiorum* was superimposed with the models from *C. jacksonii*, *C. monteithiana*, and *C.* aff. *paspali*, and different color codes were assigned to distinguish the shared and distinct regions among the proteins (Figure 6, Supplementary Figure S1; Supplementary Video S1). Overall, the comparative modeling of the six virulence genes between *S. sclerotiorum* and *Clarireedia* spp. suggested that the majority of the structural attributes were common to all isolates of *Clarireedia* spp. and *S. sclerotiorum* analyzed with only minor differences occurring at the structural level. The presence of similar sequences and identical structures is a strong indication of the regions being highly conserved amongst the two organisms, while the occurrence of distinct regions is suggestive of functional divergence among the proteins during molecular evolution (Centeno et al., 2005).

The quality assessment of the models predicted using ERRAT and VERIFY-3D programs yielded scores between 50.6667%–97.4299% and 59.27%–98.02%, respectively (Supplementary Table S4). Additionally, the PROCHECK server provided Ramachandran plot analysis results, wherein 70.8%–89.3% residues were found to be in the most favorable region, 10.4%–22.1% residues in allowed region and a few resides up to 1.7% were present in the disallowed region (Supplementary Table S4). The QMEAN Z-scores of the models ranged between −8.79 and −1.27. These model evaluation values suggest a relatively good quality of the predicted structures (Lüthy et al., 1992; Benkert et al., 2011; Messaoudi et al., 2013; Wlodawer 2017).

### Phylogeny

The phylogenetic analysis on the concatenated six pathogenicity and five conserved gene sequences (Supplementary Table S5) revealed that *C.* aff. *paspali* was more genetically related to *C. monteihiana* than to *C. jacksonii*. The NJ tree also pointed out that *Clarireedia* species were as divergent from *R. sydowiana* (CBS115975 isolate), the representative of the Rutstroemiaceae family, as from *S. sclerotiorum* (1980 UF-70 isolate), the representative of the Sclerotiniaceae family (Figure 5B). Using the divergence time of 437 Mya between *S. sclerotiorum* and *Zymoseptoria tritici* as reference (www.treetime.org), we estimated that isolates within *Clarireedia* species diverged from one another approximately 0.00, 2.99, and 0.46 Mya for *C.* aff. *paspali*, *C. montheihiana*, and *C. jacksonii*, respectively. In addition, *Clarireedia* species diverged from *S. sclerotiorum* approximately 160 Mya (Figure 5B). The molecular clock test showed that the null hypothesis of equal evolutionary rate throughout the NJ tree was rejected at a 5% significance level (*p* = 0.000E+000). The evolutionary rate between *C.* aff. *paspali*, *C. monteithiana* (*p* = 0.36917) and *C. jacksonii* (*p* = 0.09691) sequences were statistically equal. However, the evolutionary rates of *R. sydowiana* (*p* = 0.00509) and *S. sclerotiorum* (*p* = 0.00059) were significantly higher than in *C.* aff. *paspali*.

## Discussion

### Genome completion

In this study, whole genome sequencing was performed for two isolates of *C.* aff. *paspali*. H3 isolate was sequenced by coupling Illumina paired-end with PacBio approaches, while H2 isolate was sequenced with HiSeq sequencing method only. The estimated genome size of *C.* aff. *paspali* was 48.6 Mb. As this is the first draft genome for *C.* aff. *paspali*, we relied on the assembly statistics of other published *Clarireedia* genomes for comparison. Similar genome lengths (36–48 Mb) were observed for *C. monteithiana* and *C. jacksonii* (Crouch et al., 2021). The genome of *C.* aff. *paspali* H3 and H2 isolates contained a total of 193 contigs and 5 Mb contig N50 and 6,743 contigs and ∼77 kb contig N50, respectively. In comparison, the *de novo* assemblies previously published for *C. jacksonii* produced 15.63–709.08 kb scaffold N50, and 231–31,623 contigs with PacBio and/or HiSeq sequencing methods (Green et al., 2016; Crouch et al., 2021). *Clarireedia monteithiana* available genome assemblies produced 8.76–20.76 kb scaffold N50, and 15,133–3,610 contigs with Illumina HiSeq sequencing data (Crouch et al., 2021). In this study, the *de novo* assembly for *C.* aff. *paspali* H2 isolate produced a better assembly than any other published *Clarireedia* assembly sequenced with just the short reads. The bioinformatic tools used for downstream analysis have different performance and could play an important role in the quality of the final assembly (Bankevich et al., 2012). In addition, by combining Illumina HiSeq short-read and PacBio long-read sequences, the hybrid assembly approach used in this study for *C.* aff. *paspali* H3 isolate was considered effective in reducing the number of contigs and increasing the contig N50 length of the *Clarireedia* genome. Short reads provided the accuracy in the bp read and long reads, which were more error prone, gave the required read length for a better assembly. The K-mer and RepeatMasker analysis revealed that the *C.* aff. *paspali* genome has low complexity (only 2.09% of repetitive regions), similarly to other *Clarireedia* species. In addition, AUGUSTUS predicted 12,894 gene models, representing 32.32% the *C.* aff. *paspali* genome. This number is similar to the range of the gene models present in other *Clarireedia* species (range between 10,821–11,089 genes (Crouch et al., 2021). Furthermore, the completeness of assembly and annotation were evaluated by the BUSCO analysis, and an annotation completeness of 81.9% was reached. Although this study presents the most complete assembly currently available for *Clarireedia*, further studies are needed to improve the genome assemblies and provide chromosome-level reference genomes. In this draft genome, more than 91.5% of the assembly is composed in 25 contigs (>250 kb per contig). Deciphering genome continuity and assembling the large PacBio contigs into chromosomes require additional techniques such as Hi-C, which was successfully applied in genome scaffolding for a wide range of eukaryotic species (Kaplan and Dekker 2013; Wang et al., 2020). Scaffolding using Hi-C data could provide full chromosome-level assemblies for each *Clarireedia* species. However, long-reads are still needed for *C. monteithiana*. Long-reads PacBio data sequenced on Sequel SMRT-cell (as shown in this study) or nanopore data sequenced on the MinION platform (Oxford Nanopore Technologies) would allow updates to the current *C. monteithiana* genomes. Chromosome-level genomes would reveal aspects of genome evolution and would open-up a more detailed view of genome plasticity within *Clarireedia* species. Karyotype studies to visually validate the chromosome number are needed in all the *Clarireedia* species. Flow cytometry, recommended to pre-calibrate genome assembly pipelines, would allow a more accurate genome assembly (Kooij and Pellicer 2020). *C.* aff. *paspali* presented on average, the largest genome (43.9 Mb; from this study) compared to *C. monteithiana* (42.4 Mb; Sapkota et al., 2021) and *C. jacksonii* (37.5 Mb; Sapkota et al., 2021). Difference in the genome size among *Clarireedia* spp. could correspond to a difference in the chromosome number. Large structural rearrangements leading to aneuploidy were observed in several plant pathogenic fungi including the haploid hemibiotrophic plant fungus *Z. tritici*. The top 23 contigs identified in this study, representing >90% of the genome (43.8 Mbp), could constitute the chromosome-level (core and accessory chromosomes) representation of *C.* aff. *paspali* genome. With a similar genome size, *Z. tritici* (39.7 Mbp) is represented by a total of 21 chromosomes and genome plasticity was common at the population level within *Z. tritici*, where eight out the 21 chromosomes constituting the reference genome, were considered as accessory because of their presence/absence variation (Goodwin et al., 2011). Chromosome counting may also provide additional information on genetic compatibility and mating patterns allowing genetic material exchange between *Clarireedia* species. Although sexual reproduction has not been reported so far in nature in *Clarireedia* (DeVries et al., 2008), heterothallism (Liberti et al., 2012; Putman et al., 2015) and vegetative compatibility groups were observed (Powell and Vargas, 2001; Viji et al., 2004).

### Recent introduction of *C.* aff. *paspali* to the United States and genetic variation within and between *Clarireedia* species

In this study, symptoms observed in seashore paspalum on a sod farm in Hawaii presented typical signs of dollar spot. Prior to this finding, *C. jacksonii* and *C. monteithiana* were the two species identified as responsible for dollar spot in turfgrass in the United States (Salgado-Salazar et al., 2018). However, in this study, Sanger sequencing and BLAST results revealed the presence of *C.* aff. *paspali*. To date, this species has only been identified on seashore paspalum in China based on multi-locus analysis (ITS, EF, and Mcm7) (Hu et al., 2019). The major hypothesis supported by our study on the emergence of *C.* aff. *paspali* in the United States is its introduction from China rather than natural local evolution from pre-existing *Clarireedia* species. Because *Clarireedia* spp. do not produce spores, the pathogen dissemination through long-distances is most likely human-caused and attributed to movement of infected plant materials (Walsh et al., 1999; Horvath et al., 2007; Rioux et al., 2014). In addition, because *C.* aff. *paspali* H2 and H3 isolates showed similar genome sequences and HK1 sequences were 100% identical on ITS and EF, the introduction of *C.* aff. *paspali* to the United States from China must have been recent. Further dollar spot sampling in Hawaii and genomic comparison of *C.* aff. *paspali* isolates from United States and China would help us to explain *C.* aff. *paspali* emergence in the United States

At the genome level, *Clarireedia* species are closely related. *C.* aff. *paspali* isolates (H2 and H3), *C. monteihiana* isolates (DRR09 and RB-19) and *C. jacksonii* isolates (LWC-10 and HRI11) showed high genome similarity (>96%). In addition, similar evolutionary rates in all *Clarireedia* species were detected in the phylogenetic analysis on 22,983 bp of aligned sequence. A high level of synteny was observed between *C.* aff. *paspali* and the published genome of *C. jacksonii* HRI (data not shown). In addition, 25% of the orthologous clusters identified were shared by at least two *Clarireedia* species and about 16% of the orthologous clusters were common to all three *Clarireedia* species. However, phylogenetic analysis on the concatenated six pathogenicity and five conserved gene sequences, as well as the genome comparison between *Clarireedia* species showed clear differentiation between the three *Clarireedia* species. 322 orthologous groups were unique to one *Clarireedia* species. In comparison, *C.* aff. *paspali* showed the lowest fragmented and missing BUSCOs when using the fungal BUSCOs. The highest and lowest number of species-specific SNPs in the pathogenicity genes studied as well as the highest and lowest number of species-specific orthologous groups were observed for *C.* aff. *paspali* and *C. monteithiana*, respectively. In addition, three species-specific SNPs for *C.* aff. *paspali* have been identified in the ITS region and EF gene. *Clarireedia* species showed genomic variations at potential pathogenesis factors. In fact, four genes involved in morphogenesis and pathogenesis, Adenylate cyclase (*sac1*), a Type 2A Ser/Thr phosphatase B subunit (*rgb1*), a rearrangement hotspot repeat-containing protein (*rhs1*), and a polyketide synthase (*pks13*), showed presence/absence variation between *Clarireedia* species. In addition, our phylogenetic and genome comparison studies also revealed that *C.* aff. *paspali* was more closely related to *C. monteithiana* than to *C. jacksonii*. There were 2.4 times more orthogroups common between *C.* aff. *paspali* and *C. monteithiana* than between *C.* aff. *paspali* and *C. jacksonii*.

Furthermore, from within species analysis, *C.* aff. *paspali* and *C. monteithiana* showed the lowest and the highest diversity in terms of polymorphic positions, respectively. Phylogenetic analysis point-out the most recent divergence between *C.* aff. *paspali* isolates. However, DRR09 and RB-19 showed the least recent divergence (2.99 Myo). At the genome level, H2 and H3 isolates were almost genetically identical (99.51% of sequence similarity) while *C. monteihiana* isolates DRR09 and RB-19 showed the lowest genome similarity. DRR09 and RB-19 also differed by presence/absence of one gene (*pks13*) involved in appresoria formation. This pattern could be explained because *C.* aff. *paspali* isolates were sampled in the same year, on the same host and at the same location, while *C. monteihiana* isolates DRR09 and RB-19 were sampled in the Dominican Republic on seashore paspalum and in Mississippi, United States on bermudagrass, respectively.

### Pathogenicity of *Clarireedia* and its differentiation with *S. sclerotiorum* and *R. sydowiana*

Although *Clarireedia* were removed from the Sclerotiniaceae and reclassified as part of the Rutstroemiaceae family (Salgado-Salazar et al., 2018), *Clarireedia* species were found to be as genetically divergent from *R. sydowiana* as from *S. sclerotiorum*. In this study, *S. sclerotiorum* and *R. sydowiana* showed several genetic dissimilarities with *Clarireedia*. The evolutionary rate between *C.* aff. *paspali* and *R. sydowiana* or *S. sclerotiorum* were significantly different. The DNA similarity between *S. sclerotiorum* and the *Clarireedia* species was <80% on the genome level overall and 81.04% on the concatenated six pathogenicity and five conserved gene sequences studied. In addition, only seventeen of the fifty-four pathogenicity genes of *S. sclerotiorum* were identified in at least one *Clarireedia* species. However, the six pathogenicity genes compared at the protein level showed superimposition of the predicted models between *S. sclerotiorum* and *Clarireedia* spp., as well as among *Clarireedia* species. Overall, the results supported the differentiation in the virulence patterns and therefore host-range between *Clarireedia*, apparent hemibiotrophic turfgrass pathogen with its closely related necrotrophic dicto pathogen *S. sclerotiorum*. *Clarireedia* species and *R. sydowiana* differed by presence/absence of ten genes involved in pathogenesis. Three genes (*pth2*, *bi1* and *hex1*) absent in all the *Clarireedia* species but present in *R. sydowiana* may have been selectively lost in the *Clarireedia* lineage. Seven genes (*cna1*, *caf1*, *nox1*, *pac1*, *pka1* and *nacα*, and *ams2*) present in all the *Clarireedia* species but absent in *R. sydowiana* have been selected in the *Clarireedia* lineage and may play an important function in dollar spot development. In addition, more than 56% of the cytoplasmic effectors identified by EffectorP in *Clarireedia* spp. blasted neither against *Rutstroemia* spp. nor *Sclerotinia* spp. However, eight time more *Clarireedia* cytoplasmic effectors blasted against *Rutstroemia* spp. than *Sclerotinia* spp., suggesting a closer similarity of *Clarireedia* to *Rutstroemia* spp. than to *Sclerotinia* spp. in their pathogenic process.

Within the seventeen potential pathogenicity genes identified in *Clarireedia* species in this study, several similar proteins have already been confirmed in a sequencing-by-synthesis library of creeping bentgrass inoculated with dollar spot (Orshinsky et al., 2012a). In fact, based on RNA-seq analysis, two protein kinase (SHT_6900; SHT_8743:2.87), a glycerol-3-phosphate dehydrogenase (SHT_2740), an hybrid nrps pks (SHT_9252), a polyketide synthase (SHT_2432), an histidine kinase (SHT_9426), a GATA type transcription factor (SHT_2779) and two protein kinase regulator (SHT_7172 (*ste50*); SHT_4675) were either significantly down or up-regulated (>2 log fold change) during creeping bentgrass infection with dollar spot. In addition, some of these transcripts were enriched in NADPH binding domain, sac1 homology domain and polyketide synthase domain (Orshinsky et al., 2012b).

*S. sclerotiorum*, a necrotrophic fungal pathogen which can infect more than 400 plant hosts, has been demonstrated to produce oxalic acid and utilize it for successful pathogenesis (Bolton et al., 2006). Typically, for pathogens within the Sclerotinia genus, the host is infected by hyphal colonization of host tissues through stomata or wounded sections of leaves. Hyphae then exude oxalic acid into the intracellular space which lowers the pH and provides an optimal environment for pectolytic enzymes, such as polygalacturonase, to degrade cellular wall components (Heller and Witt-Geiges 2013). Initially, oxalic acid inhibits the oxidative burst within the cell and creates a reducing environment (Williams et al., 2011; Kabbage et al., 2013). After the infection is established, oxalic acid induces the production of reactive oxygen species (ROS), such as hydrogen peroxide and superoxide ions. Both these ROS’s are messengers that initiate cell death (Williams et al., 2011). The possibility that oxalic acid is also involved in pathogenicity of *Clarireedia* spp. was first raised following its detection in the pathogen grown in pure culture (Venu et al., 2009). Later, other studies showed the increase in oxalic acid content in dollar spot infected turfgrass (Orshinsky et al., 2012a; Rioux et al., 2020). Although oxalic acid was identified as an important pathogenicity factor in species of *Sclerotinia*, in this study, only two (*pac1*, *nox1*) out of the six genes involved in oxalic acid production (*odc2*, *oah*, *sod1*, *pth2*) in *S. sclerotiorum* have orthologs in *Clarireedia* species. Recently, Townsend and colleagues (Townsend et al., 2020) suggested that *C. jacksonii* could potentially present a different pathogenesis than *S. sclerotiorum*. In addition, beside *nox1*, another gene (*nox2*) belonging to the NOX family of NADPH oxidases has been identified in *Clarireedia* species. At the genomic level, *nox1* and *nox2* carried 27 and 23 species-specific SNPs respectively; however NOX1 and NOX2 proteins were not polymorphic between *Clarireedia* species and presented a high identity with *S. sclerotiorum* proteins, supporting its important role in the virulence and the wide host range patterns of *Clarireedia* spp. NOX family enzymes are critical sources of cellular superoxide anions and ROS *via* electron transfer. These enzymes contribute to a wide range of physiological functions including pathogenicity in several necrotrophic fungi with a wide host range from the Ascomycota division, such as *Magnaporthe oryzae* (Egan et al., 2007), *Botrytis cinerea* (Segmüller et al., 2008), *Verticillium dahliae* (Zhou et al., 2017), *Claviceps purpurea* (Giesbert et al., 2008), *Alternaria alternata* (Yang and Chung, 2012) and *Sclerotinia sclerotiorum* (Kim et al., 2011). NOX family enzymes have also been shown to be involved in sclerotia formation and development of fungal multicellular structures in *S. sclerotiorum* (Kim et al., 2011). These two nox genes could have distinct cellular functions in the pathogenicity of *Clarireedia* spp. For example, *nox1* was shown to regulate fungal proliferation within plant tissues while *nox2* is necessary for the penetration of host cells through the cuticle in *M. grisea*, *V. dahliae*, and *B. cinerea* (Segmüller et al., 2008; Egan et al., 2007; Giesbert et al., 2008; Ryder et al., 2013; Zhou et al., 2017). Typically during infection, mycelium of *Clarireedia* spp. penetrates the plant directly *via* the cuticle by forming an appressorium or indirectly through cut leaf tips and natural openings (stomata), grows to invade plant tissues and at a later stage forms fungal stroma in dead tissue to survive from one season to the next (Rioux et al., 2014). In addition, nox1 was shown to regulate differentiation of sexual structures (cleistothecia, perithecia development and ascospore production) in several fungi (Lara-Ortiz et al., 2003; Lalucque and Silar, 2003; Cano-Dominguez et al., 2008; Wang et al., 2014). Although, in the case of *Clarireedia* spp., no sexual reproduction has been observed in nature so far (DeVries et al., 2008), mating type, genetic diversity and linkage disequilibrium studies pointed-out the potential for occurrence of the sexual cycle in *Clarireedia* (Hsiang and Mahuku 1999; Liberti et al., 2012; Putman et al., 2015).

Furthermore, three other pathogenesis genes (*pka1*, *smk3* and *ste12*) involved in mitogen-activated protein kinase (MAPK) cascade have been identified in *Clarireedia* spp. MAPK are involved in fungal pathogen responses to host and environmental stimuli and several are conserved in plant pathogenic ascomycetes (Jiang et al., 2018). *pka*, protein kinases, such as *pka1* identified here, participate in surface recognition in the early stages of the MAPK cascade during plant infection. *smk3* is an ortholog of *stl2* in yeast. *stl2* is a conserved gene in all plant pathogenic filamentous ascomycetes regulating cell wall integrity and pathogenesis (Turrà et al., 2014). In *Sclerotinia sclerotiorum*, *smk3* was shown to be involved in sclerotia formation and aerial hyphal growth (Bashi et al., 2016). Its ortholog (*mps1*) in *M. grisea* is essential for early stages of appressorium development (Li et al., 2012). Thus, because *Clarireedia* spp. is an appressorium-forming pathogen, *smk3* could play an important role in its pathogenesis. In addition, *ste12*, a downstream transcription factor of MAPK pathway of yeast fus3 and kss1, is also essential for plant infection in pathogenic fungi. With the deletion of *ste12*, *M. oryzae* was unable to infect and develop appressorium for plant penetration (Li et al., 2012). Other genes identified here and potentially involved in appressorium formation were *pks13*, *caf1*, *ams2*, *rgb1*, and *rhs1*. *caf1* presented several species-specific SNPs for each *Clarireedia* species but only 1 SNP, identified in *C*. aff. *paspali*, was non-synonymous and non-conservative. Similarly, four and one non-synonymous and non-conservative SNPs were identified in *ams2* for *C*. aff. *paspali* and *C. monteithiana*, respectively. *pks13* was absent in *C*. aff. *paspali* but present in *C. jacksonii*. *rgb1* and *rhs1* were exclusively present in both isolates of *C. monteithiana* and *C*. aff. *paspali*, respectively. Additional studies are needed to evaluate the contribution of these genes and their SNP to the variation in gene function and virulence between *Clarireedia* spp.

In addition, *gpd*, a glyceraldehyde-3-phosphate dehydrogenase (*gpd*), was also identified in all three *Clarireedia* species. GAPDH is a key enzyme in the glycolytic pathway that plays a significant role in pathogenesis of filamentous fungi (Barbosa et al., 2006; Egea et al., 2007). *gpd* transforms the glyceraldehyde 3-phosphate to glycerate-1, 3-biphosphate, generating the production of NADPH. *gpd* is generally present as a single copy (Liao et al., 2008), is highly conserved in filamentous fungi and is typically used for species identification (Ghuffar et al., 2018; Huang et al., 2018; Torres-Calzada et al., 2018). In this study, *C*. aff. *paspali*, *C. monteithiana* and *C. jacksonii* presented ten, seven and seven species-specific SNPs within the *gdp* gene that could be used for species identification, respectively.

## Conclusion

In summary, we report the first draft genome of *C*. aff. *paspali*. This genome presents the most complete annotation among *Clarireedia* species so far and should be considered as the reference for future genomic and proteomic studies. This is also the first report of *C*. aff. *paspali* in the United States Several genes potentially involved in *Clarireedia* morphogenesis and pathogenesis were highlighted from this genomic analysis. Phylogenetic analysis revealed genetic differentiation among *Clarireedia* species. Because of the limited genomic resources published for members of the Rutstroemiaceae family and for *Clarireedia* species more specifically, our study provides valuable genomic information for future research discoveries on plant pathogens.

## Data Availability

The two *C.* aff. *paspali* isolates, H2 and H3, were deposited in GenBank under the BioSample SAMN22087743 (H2) and SAMN22083495 (H3). In addition, the ITS, TUB, CaM and EF sequences of the two *C.* aff. *paspali* isolates were deposited in GenBank: MZ578438, MZ620645, MZ620646, and MZ620647 for H2 isolate, respectively; MZ578439, MZ620643, MZ620644, and MZ620642 for H3 isolate, respectively. Raw reads of this Whole Genome Shotgun sequencing project were deposited at GenBank as Sequence Read Archive (SRA) under the BioProject PRJNA769080. Free access to the raw reads and the assembled genomes and annotation is also available through https://bahrilab.caes.uga.edu (under Genomic data tab).

## Supplementary material

SUPPLEMENTARY VIDEO S1 Movie animations of the predicted protein structures of six virulence genes from *S. sclerotiorum* with *Clarireedia* spp. showing the structural similarities and differences at the protein level.
