# Supplementary material for: Whole genome sequencing of Clarireedia aff. paspali reveals potential pathogenesis factors in Clarireedia species, causal agents of dollar spot in turfgrass
Source: Front Genet. 2023 Jan 5;13:1033437. doi: 10.3389/fgene.2022.1033437 (PMC9849252; doi:10.3389/fgene.2022.1033437)
Supplement: Supplementary file 2 [file Presentation1.pdf]

APPENDIX

Supplementary Protein Structure Videos. Movie animations of the predicted protein structures of the following six virulence genes from *S. sclerotiorum* with *Clarireedia* spp. showing the structural similarities and differences at the protein level:

1. *gadph*
2. *nox1*
3. *nox2*
4. *pka*
5. *pph1*
6. *smk3*
